# Epitranscriptome: Review of Top 25 Most-Studied RNA Modifications

**DOI:** 10.3390/ijms232213851

**Published:** 2022-11-10

**Authors:** Viktoriia A. Arzumanian, Georgii V. Dolgalev, Ilya Y. Kurbatov, Olga I. Kiseleva, Ekaterina V. Poverennaya

**Affiliations:** Institute of Biomedical Chemistry, 119121 Moscow, Russia

**Keywords:** RNA modifications, epitranscriptome, RNA

## Abstract

The alphabet of building blocks for RNA molecules is much larger than the standard four nucleotides. The diversity is achieved by the post-transcriptional biochemical modification of these nucleotides into distinct chemical entities that are structurally and functionally different from their unmodified counterparts. Some of these modifications are constituent and critical for RNA functions, while others serve as dynamic markings to regulate the fate of specific RNA molecules. Together, these modifications form the epitranscriptome, an essential layer of cellular biochemistry. As of the time of writing this review, more than 300 distinct RNA modifications from all three life domains have been identified. However, only a few of the most well-established modifications are included in most reviews on this topic. To provide a complete overview of the current state of research on the epitranscriptome, we analyzed the extent of the available information for all known RNA modifications. We selected 25 modifications to describe in detail. Summarizing our findings, we describe the current status of research on most RNA modifications and identify further developments in this field.

## 1. Introduction

The discovery of the first modification of RNA occurred in 1957, when the “fifth nucleotide”, pseudouridine, was identified [1]. By 1995, 93 naturally occurring modified nucleosides were identified primarily in two large classes of RNA, rRNA and tRNA. However, knowledge of the exact frequency and function of the majority of these modifications was obscure due to the limits of the available technology [2]. In the last 20 years, with the advent of technologies for next-generation sequencing (NGS) and improvements to liquid chromatography–mass spectrometry (LC-MS/MS), more than hundreds of various modifications have been characterized across all RNA species and domains of life [3].

It is now widely appreciated that RNA modifications are significantly more numerous and common in cellular RNA than thought initially—The current release of the main database for cataloging RNA modifications, MODOMICS, lists slightly more than 300 such modifications [4]. These modifications can be relatively simple, involving, for instance, only an addition of a methyl group, or complex, as is the case with 5-methoxycarbonylmethyl-2-thiouridine, which is installed in substrate RNA in a multistep process by a group of enzymes [5]. Despite the large number of known RNA modifications, only a few of the most popular ones continue to be the subject of general reviews on this topic.

Many RNA modifications are indispensable for the function of RNA species. They are required for proper folding [6], transcription [7], splicing [8], translation [9], RNA transport [10], and immune responses [11]. Many such modifications are always present in RNA in specific positions. However, it is being increasingly reported that some RNA modifications are dynamic, affecting the behavior of RNA molecules in a temporal and context-sensitive manner [5]. While this phenomenon has only recently been discovered, such dynamic behavior has already been associated with important cellular processes, for instance, response to heat shock [12] or different toxins [13].

The significance of the research on RNA modifications is highlighted by their tight association with diseases of various natures [14]. Particular attention has been devoted to the role of modifications in cancer, where disbalances of RNA modification levels are often linked to a more severe prognosis [15]. Aberrant RNA editing has been associated with hepatocellular carcinoma (HCC) [16], Hodgkin lymphoma [17], and many other types of cancer [18]. On the other hand, the recent success of mRNA-based vaccines against SARS-CoV-2 is mainly due to the N1-methylpseudouridine modification that increases the translational efficiency of vaccinal mRNA [19].

The presented facts signify the importance of research on RNA modifications, but most of the attention in recent years has been paid to only a handful of well-established modifications. At the same time, our knowledge of other modifications is rapidly growing. Annotating modifications with data on their distribution and function is critical to obtain the global picture of the role and prevalence of RNA modifications. Accordingly, in this review, we analyzed the popularity of known modifications as evidenced by the number of relevant articles and provided a comprehensive description of the frequency of occurrence, distribution, biological function, connection to diseases, and detection methods for 25 modifications. Together, this information paints the role of RNA modifications as widely occurring in all RNA classes from all domains of life and as key players in cellular processes.

## 2. Current Status of Research on RNA Modifications

To understand the current level of research on RNA modifications in total and per individual RNA modifications, we used the full name of each modification provided in the MODOMICS database [4] together with the keyword “RNA” via the Entrez API to estimate the number of articles in the PubMed database mentioning the modification name in the title or the abstract. We then manually reviewed the results starting from the top (RNA modifications with the most articles) and removed modifications that were either incorrectly interpreted by the Entrez search engine (indicated by a significantly smaller number of articles when querying PubMed directly) or modifications primarily investigated outside the context of endogenous cellular RNA, for example, 4-thiouridine, which is a major part of multiple NGS-based techniques [20]. As a result, we discovered 45 modifications that had ten or more relevant articles (Table S1). Since we used strict search criteria to minimize the number of irrelevant findings, our figures likely represent a conservative estimate. They do not include all publications related to RNA modifications.

**Figure 1:**
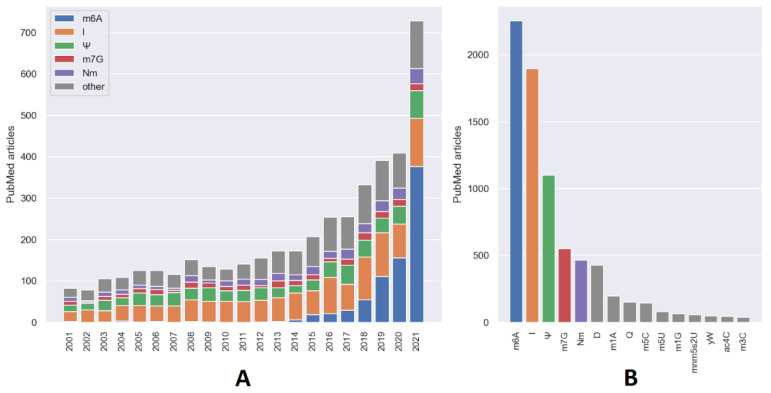
(**A**) Number of published articles that are strongly related to specific RNA modifications in the PubMed database per year. (**B**) Total number of articles on specific RNA modifications in the PubMed database.

The research on RNA modifications is an actively developing area, as evidenced by a continuous increase in the number of relevant articles in recent years (Figure 1). Our search strategy correctly identified the most well-established RNA modifications as the modifications with the largest number of relevant articles, both per year (Figure 1A) and in total (Figure 1B). The modification with the most attention growth in recent years is N6-methyladenosine, which is not surprising considering its abundance in mRNA, its now-established strong connection to cancer processes, and a plethora of high-throughput methods for its detection (see below). While other modifications demonstrated more negligible growth, it is noteworthy that the number of articles related to modifications outside the top five has been growing in recent years (Figure 1A).

## 3. RNA Modifications at Large

To provide a justified overview of the current state of the characterization of individual RNA modifications, we selected the top 25 RNA modifications with the most relevant articles in our list for further characterization. We collected all the available information regarding their frequency of occurrence, distribution in RNA, established biological function, association with various diseases, and specific methods designed to detect them in RNA (Table 1). Our goal was to spotlight not only the well-established modifications, but also the less popular ones that are usually omitted from most general reviews, despite the recent indications of their vital biological roles and frequent occurrence in endogenous RNA.

Table 1 reveals that selected modifications affect distinct bases in similar proportions, which aligns with the data for all known modifications [4]. Nucleotides are modified by specific enzymes such as RNA-modifying proteins (RMPs): writers, erasers, and readers. RMPs modify nucleotides, changing their properties and functions. Modifications to nitrogenous bases can either stabilize or destabilize the base pairs to trigger refolding into more stable structures or weaken them. The influence picture of modifications on RNA structures is only just beginning to develop due to a lack of methods. The existing experimental methods (X-ray crystallography and NMR spectroscopy) are resource-intensive, and there are not many efficient computational methods that are able to predict complex motifs or even three-dimensional conformations of RNA from sequence data [118].

In terms of the occurrence of RNA modifications across domains of life, most modifications are present in all three domains with a few exceptions, for instance, archaeosine, which is detected only in archaea [102]. This is consistent with the theory that modified RNA existed well before the advent of cellular life [119].

The majority of modifications from Table 1 can be found in transfer RNA, the most heavily modified class of RNA—each tRNA molecule on average has 13 modifications, many of which are required for proper codon decoding and robust translation [9]. Ribosomal RNA is also frequently modified, albeit to a lesser extent than tRNA [120]. Since tRNA and rRNA are the most abundant RNA classes, most known modifications were identified in them [121]. However, thanks to improvements in enrichment strategies and sequencing techniques, many of the modifications that were considered exclusive to rRNA and tRNA, as well as other new modifications, are now found in other RNA classes, including mRNA [122,123], miRNA [124], lncRNA [32], and even short-lived species such as eRNA [125].

In terms of importance, the selected modifications can be firstly classified as essential and nonessential. Essential modifications are shown to be necessary for important cellular processes, for example, translation, as is the case with dihydrouridine or 1-methylguanosine, for instance (see text below for all examples and details). Generally, a lack of these essential modifications significantly impairs cellular growth or causes death. We estimate that 10 modifications from our list (40%) represent essential modifications. It is possible that this percent is higher, since some of the nonessential modifications could have undiscovered essential roles. An additional indicator of the importance of a modification is its connection to human disease. Eleven of the modifications (44%) from our list are associated with various diseases, and nine of these (36%) are associated specifically with cancer. These results highlight the necessity of studying the epitranscriptome in detail, since RNA modifications are frequently essential for proper growth or linked to ailments of various natures.

As for the methods for the detection of RNA modifications, they are impressively numerous and range from polymerase chain reaction (PCR) and mass spectrometry to RNA sequencing and even fluorescence in situ hybridization (FISH). In the table, the most popular methods are given for each modification, but this is an extensive and complex topic that goes beyond the scope of this review. For excellent coverage of the range and specifics, as well as the technical challenges of the methods for the detection of RNA modifications, readers can refer to the review by Huang et al. [126].

## 4. RNA Modifications in Depth

Below, we describe the properties of several groups of biochemically and functionally related modifications, emphasizing the recent progress in establishing unique features that affect their distribution in RNA and their molecular function, as well as the specifics of the detection methods.

### 4.1. N6-methyladenosine (m6A)

N6-methyladenosine (m6A) is the most-studied modification in all life domains [21]. m6A is prevalent in eukaryotic RNA, especially within higher eukaryotic cells. m6A sites are present in mRNA, tRNA, rRNA, circRNA, miRNA, snRNA, and lncRNA [127]. The m6A modification is abundant in mRNA, and is essential for regulating RNA splicing, translation, stability, translocation, and the high-level structure [127,128].

In eukaryotes, the m6A modification is located in all mRNA regions: 5′UTR, CDS, and 3′UTR. The modification residues were enriched in 5′-UTRs around the stop codons and in 3′ UTRs adjacent to the stop codons. Approximately 35% of m6A residues are located within the CDS [129]. This modification is enriched not only in eukaryotes, e.g., in *A. thaliana*, but the m6A sites are also detected near the start codons [129]. The m6A peaks were frequently detected at miRNAs target sites [130].

N6-methyladenosine is installed by “writer” proteins, including METTL3, METTL14, METTL16, WTAP, KIAA1429, ZCCHC4, RBM15, ZC3H13, and ZCCHC4 [131,132]. In addition, the m6A reader proteins can recognize m6A-modified RNAs. The first group of proteins regulates the alternative splicing or processing of target transcripts and includes such enzymes as YTHDF1/2/3, YTHDC2, HNRNPC, and HNRNPA2B1 [133]. The second group includes eIF3 in eukaryotes and regulates protein expression [134]. The m6a modification is one of the possible reversible modifications due to the enzyme-easers FTO и ALKBH5 [27].

The landscape of m6A in RNA was first uncovered by NGS. Nowadays, m6A can be detected by multiple methods, including m6A-seq [135], MeRIP-seq [25], miClip [136], PA-m6A-seq [137], and m6A-CLIP [138]. With the development of third-generation sequencing technologies, m6A can be detected by Oxford Nanopore technology—direct RNA-seq [23].

N6-methyladenosine is involved in multiple human diseases, including obesity, heart failure, and especially cancers [18,26]. m6A regulators may also serve as a potential target in therapeutic strategies [139].

### 4.2. Inosine (I) and 1-methylinosine (m1I)

Adenosine-to-inosine editing is a major pathway of RNA modifications that is present in all domains of life [140]. Inosine has different hybridization rules than adenosine; thus, A-to-I editing can affect the local RNA structure and the interactions with other RNA molecules and proteins [29]. Inosine is found in tRNA and mRNA as well as other noncoding RNA, such as miRNA [141].

In tRNA, the adenosine at position 34 (the first base of the anticodon) is frequently modified to inosine to support the mechanism of wobble codon recognition, since inosine can hybridize with adenosine, guanosine, and uridine [28]. This modification is absent in archaea and usually only found in tRNA^Arg^ in bacteria, while eukaryotes have seven or eight tRNAs modified in this way [142]. This modification is installed by the enzyme TadA in bacteria and adenosine deaminase acting on tRNA (ADAT) in eukaryotes [28]. Additionally, inosine has been discovered in positions 37 (the base directly adjacent to the anticodon) [143] and 57 (only in archaea) [144] in the form of 1-methylinosine, but the function of these modifications is not yet clear [117].

In mRNA, A-to-I editing changes the hybridization rules, since inosine is interpreted by tRNA and RNA-binding proteins (RBPs) as guanosine [145]. The mRNA editing in eukaryotes is performed by a family of enzymes called adenosine deaminases acting on RNA (ADARs), which are conserved across the Animalia kingdom [146]. Interestingly, the editing only occurs in double-stranded regions of mRNA and can affect the three-dimensional structure of the transcript [147]. In noncoding regions of mRNA, A-to-I editing can lead to an altered stability and localization of the transcripts [148]. In coding regions, A-to-I editing can change the codon identity, protein sequence, and function [145]. For instance, the glutamate receptor subunit GRIA2 has a transmembrane channel that can lose permeability to Ca^2+^ as a result of A-to-I editing in its coding region of mRNA [149]. Notably, A-to-I editing frequently affects brain-specific proteins [150].

Standard NGS protocols can reveal the locations of A-to-I editing, since inosine is interpreted as guanosine, and A-G genome mismatches arise in the final result [141]. Recently, a method to detect inosines in full-length transcripts using nanopore sequencing has been presented [33]. Additionally, adenosine-to-inosine RNA editing can be detected via LC-MS/MS with a high sensitivity [151,152].

The A-to-I editing mechanism has been repurposed in the last three years to introduce precise changes to specific mRNAs by a complex of ADAR and RNA-targeting catalytically dead Cas nucleases [153]. This could lead to new effective therapy options for multiple severe disorders [154].

### 4.3. Pseudouridine (Ψ)

*Pseudouridine* (Ψ) was the first modified ribonucleoside discovered in 1951 [155] and is the most abundant post-transcriptionally modified nucleotide in RNA from all three domains of life [34]. It is detected in a wide range of RNA, from tRNA and rRNA to various snRNAs [34]. In tRNA, Ψ is detected at position 55 and is associated with stress resistance [156]. In *E. coli*, this modification is catalyzed by the truB gene [157]. In archaeal tRNA, it is installed by Cbf5 and Pus10 [158], and in Eukaryota, it is installed by Pus4 [159]. Pseudouridylation also increases the stability of tRNA [160]. In rRNA, the Ψ sites are crucial for ribosome assembly and protein synthesis [161].

Uridine is transformed into Ψ by a class of enzymes known as pseudouridine synthases. For each species, these enzymes are different. For instance, in humans, Ψ is installed by a methyltransferase complex, including RPUSD1/2/3/4, PUS10, PUS7L, and TRUB2, while in yeast, this complex includes PUS2/5/6/8/9 enzymes. Both species have similar pseudouridine synthases, for example, PUS1/4 and DKC1 [34]. Pseudouridylation is irreversible due to the conversion from U to Ψ, as the C-N glycosidic bond of U is isomerized to a much more inert C-C bond in Ψ. This modification can be detected by multiple NGS protocols, such as Pseudo-Seq [36], PSI-seq [37], CeU-Seq [38], Ψ-Seq [39], and RBS-seq [40]. Pseudouridylation has been implicated in various human diseases, including multiple primary cancers [35,162], mitochondrial myopathy, sideroblastic anemia [163], and dyskeratosis congenita [41].

### 4.4. 5-methyluridine (m5U)

The next uridine modification is 5-methyluridine (m5U), having a single methyl substituent at the 5-position on the uracil ring. It is frequently found in bacterial and eukaryotic tRNAs at position 54 in the T loop [73]. m5U sites were recently detected in human mitochondrial tRNAs. The 5-methyluridine modification is also found in rRNA, such as the 23S rRNA at positions U747 and U1939 in *B. subtilis* [164,165]. The modification increases the fidelity and efficiency of protein synthesis by stabilizing the three-dimensional structure of tRNA in vitro [166]. Experimental methods such as FICC-Seq [70], miCLIP-seq, and iCLIP [71] can be used for m5U detection.

In *E. coli* and *S. cerevisiae*, 5-methyluridine formation is catalyzed by various types of enzymes such as TrmA [167] and Trm2p [168]. In mammalian cells, m5U is produced by the TRMT2A and TRMT2B enzymes [169]. This modification is associated with breast cancer [166] and systemic lupus erythematosus [72].

### 4.5. 2′-O-methylation of ribose (Nm or 2’-O-Me)

In short, 2′-O-methyl ribose is Nm, where N stands for any nucleotide. Such a modification can occur on any base by adding a methyl group (-CH3) to the 2′-hydroxyl (-OH) of the ribose moiety. Nm modifications (Am, Gm, Um, and Cm) are found in multiple types of RNA, such as snRNA, snoRNA, lncRNA, rRNA, tRNA, and mRNA [170]. 2′-O-methylation is frequently located at position four in tRNA [171], but most commonly at position seven in eukaryotes and positions 4, 18, 32, 34, 39, 44, and 54 in bacteria. Positions 4, 18, 32, 34, and 44 are found in *S. cerevisiae* [172]. The modification occurs on the 5′ and 3′ UTRs in mRNA and also on AGUA-motif in CDS [77]. Such a modification has the potential to influence the RNA structure, stability, and interactions. For instance, it can disrupt RNA tertiary structures. The modification plays a role in epigenetic gene regulation [74].

The Nm modification is produced by fibrillarin in eukaryotes and their homolog Nop1 in yeast [173]. Different enzymes produce the Nm modification for different species and nucleotides. For example, in yeast, the Am modification is catalyzed by the Nop and Trm13 enzymes. In addition, this modification is produced in *Streptomyces actuosus* by Nsr [174]. Cm is catalyzed by Trm13, Nop1, and Trm7 in yeast and RlmM and RsmI in *E. coli* [175].

The modification can be detected by various methods, from liquid chromatography coupled with LC/MS and two-dimensional thin-layer chromatography (2D-TLC) to high-throughput sequencing (Table 1) [76,77,79].

2′-O-methylation is involved in multiple human diseases, such as Prader–Willi syndrome, asthma, Alzheimer’s disease [74], and breast cancer [176].

### 4.6. 1-methyladenosine (m1A)

As another modification that involves the methylation of adenosine, in this case at N1, 1-methyladenosine is known to play an essential structural role in tRNA and rRNA in all organisms [177]. The presence and relevance of m1A in other RNA classes, primarily mRNA, is under serious doubt despite initial findings (see below).

In tRNA, m1A has been identified at a total of six positions, one of which, m1A58, is conserved across all three domains of life [178]. Other positions are less universal and are often domain-specific or found only in mitochondrial tRNA [178]. In humans, the m1A58 modification of cytosolic tRNA is installed by the heterotetramer complex TRM6-TRM61A, and a knockdown of these proteins is detrimental to the growth of human cells [179]. This is explained by the critical role of m1A58 in the stabilization and maturation of eukaryotic initiator-tRNA^Met^ [6]. In other tRNAs, m1A58 can be present at various levels and can even be demethylated by the protein ALKBH1, which, however, can lead to a decreased usage of the tRNA [180]. Interestingly, increased expression and activity of TRM6-TRM61A complex are associated with the progression of hepatocellular carcinoma [54]. Another location of 1-methyladenosine in tRNA at position nine is commonly observed in animal mt-tRNA, where a TRM61B enzyme installs it. At this position, m1A is essential because it is required for the correct folding of mt-tRNA [181].

N1-methyladenosine is structurally vital in ribosomal RNA [177]. In the 28S rRNA in human cells, m1A1322 is installed by the protein nucleomethylin (NLM), and this position is conserved in yeast and *C. elegans* [182]. The loss of NLM leads to the impaired folding of the large ribosomal subunit and causes p53-dependent cell death in humans [183], as well as significantly decreased viability in mice [184].

As for mRNA, two initial studies utilizing an enrichment strategy with an m1A-specific antibody and subsequent RNA-sequencing identified large numbers (1000 for m(1)A-ID-seq [185] and 7000 for m1A-seq [59]) of m1A sites in human mRNA, located primarily in 5′UTR. However, subsequent studies utilizing more precise methods such as m1A-seq-TGIRT [58] and m1A-MAP [186] detected significantly fewer high-confidence sites of m1A in mRNA. The reanalysis of the two initial studies revealed that most of the identified sites were due to the cross-reactivity of the antibody used for the IP experiments [187]. Therefore, while a m1A modification can, in principle, occur in mRNA, its frequency and stoichiometry are low, and its role remains to be further investigated.

### 4.7. Dihydrouridine

Until recently, dihydrouridine (D) was found only in transfer RNA, located in the appropriately named D loop, and very seldom in prokaryotic rRNA [49]. However, several articles published last year reported dihydrouridine’s occurrence in mRNA, lncRNA, and snoRNA, providing evidence for its physiological importance (see below).

In tRNA, dihydrouridine is found at positions 16, 17, 20, 20a, 20b, and 47, although the specific positions may vary from species to species [188]. Positions 16 and 20, located in the T loop of transfer RNA, are the most frequently edited since dihydrouridine at these positions is essential for the correct folding of tRNA [189]. In humans, dihydrouridine is installed by dihydrouridine synthases 1–3 (DUS1-3) [49], whose altered expression has been implicated in the progression of non-small-cell lung carcinomas (NSCLC) [190].

Two recently developed NGS-based methods have revealed the existence of dihydrouridine in the mRNA, lncRNA, and snoRNA of yeast and human cells. The first method, Rho-seq, utilizes rhodamine labeling to detect dihydrouridine transcriptome-wide [52]. Besides the established positions of D in tRNA, this method led to the identification of 143 dihydrouridine sites in *S. pombe*, which were spread across 125 protein-coding genes and one lncRNA. It is reported that the modification of tubulin mRNA is essential for proper meiotic chromosome segregation and gamete viability. D-seq, another method developed to uncover the distribution of dihydrouridine in the transcriptome, was used to identify 48 novel D sites in 23 different snoRNAs and 130 sites in 112 mRNAs of *S. cerevisiae* [51]. Together, these results show that dihydrouridine is present in more RNA classes than originally thought and has important biological roles.

### 4.8. N4-acetylcytidine (ac4C)

N4-acetylcytidine (ac4C) is one of the most-studied modifications of tRNA and rRNA and is found in all domains of life [92]. A recent study also illustrated that this modification is detected in mRNA [94]. N4-acetylcytidine was found in position 12 of the yeast leucine tRNA (tRNA^Leu^) [191] and the brewer’s yeast serine tRNAs (tRNA^Ser^) [192]. This modification maintains the stability of tRNA^Ser^ and helps to increase the high fidelity of protein translation [193]. In human and yeast rRNA, the ac4C sites were detected in helixes 34 and 45 [92]. These positions are essential for maintaining the accuracy of protein translation [194]. In mRNA, ac4C is enriched in the CDS and 5′ UTR [94]. N4-acetylcytidine increases mRNA stability and protein translation efficiency [195]. Remarkably, the ac4C sites are absent in the mRNA from HEK293 cells [93], but present, for instance, in human HeLa cells [94].

N-acetyltransferase 10 (NAT10), a member of the GCN5-related NAT (GNAT) family of histone acetyltransferases, catalyzes the formation of ac4C on rRNA, tRNA, and mRNA [196]. Additionally, the formation of ac4C occurs with the help of the ribosomal RNA cytidine acetyltransferase 1 (Rra1) in rRNA, tRNA, and mRNA [94,95]. The tRNA acetyltransferase (TAN1) also plays an important role in the formation of ac4C on tRNA^Leu^ and tRNA^Ser^ in *S. cerevisiae* [197].

Nowadays, ac4C can be detected by various methods, including LC-MS/MS [198] and acRIP-seq [199].

### 4.9. Methyluridine (m3U)

3-Methyluridine (m3U, N3-Methyluridine) can be detected in the 23S rRNA of archaea; 16S and 23S rRNA of eubacteria [114]; and 18S, 25S, and 28S rRNA of eukaryotes [113]. The 28S rRNA contains this modification at position 4513 [114] and the 16S rRNA of *E. coli* contains it at position U1498 [200]. The yeast 25S rRNA contains two m3U residues at positions 2634 and 2843. These modifications are located in domain V of the 25S rRNA, and are responsible for the catalytic function of the ribosome [201]. The Bmt5 (Yil096c) and Bmt5 (Ylr063w) enzymes are responsible for the methylation of uridine residues in positions U2634 and U2843. 3-Methyluridine is present in tRNA at position U32 [202]. This modification is converted by RsmE (YggJ), which belongs to a newly discovered family of uridine MTases [203]. The m3U sites could play a role in either intersubunit associations or tRNA selection due to the residues being close to the 23S rRNA and P-site tRNA anticodon [204]. Additionally, 3mU in single-stranded RNA could be demethylated by the FTO enzyme [113,205].

Methylation-sensitive RNA fluorescence can detect m3U in in situ hybridization (MR-FISH), which is sensitive to single methylations, and can characterize the composition of heterogeneous mixtures of cells that differ only in their RNA methylation [85]. In addition, this modification can be detected by reversed-phase high-performance liquid chromatography (RP-HPLC) [114].

### 4.10. 5-methylcytidine (m5C) and 5-formylcytidine (f5C)

The methylation of the cytosine C5 atom has been identified in all three domains of life and many RNA types—aside from tRNA, rRNA, and mRNA, m5C is present in many classes of other noncoding RNAs [206].

In rRNA, m5C is found in small numbers that differ from one species to another [207]. For instance, *E. coli* rRNA only contains three m5C residues, while *T. thermophilus* has five or six [207]. In human cells, the 28S rRNA contains two m5C sites, m5C3761 and m5C4413, and it is reported that they are indispensable for the stable folding of rRNA and ensuring translational fidelity [208]. These sites are well-conserved in eukaryotes [209].

m5C has also been identified in tRNA, where it is usually located at several positions in the variable loop, anticodon loop, and T stem [189]. Two enzymes, NSUN2 and TRMDT1, are responsible for installing this modification in different subsets of tRNA in humans [210]. In these positions, 5-methylcytidine is believed to ensure proper tRNA folding and stability, codon–anticodon interactions, and the maintenance of the reading frame [211]. In several tRNAs, for example, in human mt-tRNA^Met^, C34 is modified to 5-methylcytidine and subsequently to 5-formylcytidine, which is required for the proper decoding of the AUA codon in mitochondria [68]. The insufficient NSUN2-mediated deposition of methyl groups on the cytidines in tRNA has been linked to neurodevelopmental disorders and oncogenesis [15,212]. Remarkably, some of the less crucial m5C sites in tRNA can be written/erased dynamically, conferring tolerance to various toxic exposures [13].

While the existence of 5-methylcytidine in mRNA has been known for the last 60 years, the distribution frequency and the exact locations of this modification have remained largely unknown until the last decade, when a variety of NGS-based methods were developed for this task [213]. m5C has been shown to be present in bacterial, archaeal, and eukaryotic mRNA [214]. However, the initial studies investigating the locations of m5C modifications in mRNA have produced different and somewhat controversial results regarding the frequency of this modification, with the total number of identified sites varying from 745 [121] to more than 10,000 [210]. Recently, an improved technique based on bisulfite sequencing was applied to discover m5C locations in human and mouse mRNA, resulting in the identification of a total of 3212 and 2498 high-confidence sites, respectively, with the highest density of m5C observed in the 5′UTR [215]. In mRNA, 5-methylcytidine has been shown to affect its stability [65], translation rate [216], and potential to form double-stranded structures [217].

The m5C modification has also been found in other classes of RNA, such as lncRNA, where it can increase its stability [218]; vault RNA, where it is required for its biogenesis [219]; and other noncoding RNA [220].

### 4.11. 7-methylguanosine (m7G)

7-methylguanosine is a common and well-known RNA modification found in all studied organisms [42]. Its recognition is associated mainly with its role as a part of the eukaryotic mRNA cap structure, a prominent feature of eukaryotic mRNA required for the proper translation and evasion from the innate immune system [221]. However, here we would like to focus on the roles of internal m7G modification.

Internal m7G has been detected in rRNA, tRNA, mRNA, and miRNA [42,43]. Ribosomal RNA from bacteria and eukaryotes contains several 7-methylguanosines [46]. In humans, SSU rRNA is methylated at position G1639, and this modification is conserved in yeast [222]. However, the methylation of this position by methyltransferase METTL2 is apparently not vital [222]. In contrast, at position 46 in the variable region of tRNA, m7G is present almost universally across all domains of life, and its biogenetic, structural, and functional properties have been described in greater detail [42].

Several high-throughput methods aimed at detecting m7G modifications with a single-base resolution have been recently proposed. The first method, m7G-meRIP-seq, resulted in the detection of 3823 m7G peaks in mRNA shared between three human cell lines [44]. Shortly thereafter, the m7G miCLIP-seq method was utilized to detect m7G sites in the HeLa and Hek293 cell lines [45]. The identified number of m7G locations (2896 internal m7G clusters within 1635 mRNAs in HeLa cells and 4522 clusters within 2318 mRNAs in 293T cells) was similar to a previous study, although there were differences in the exact positions of m7G in transcripts between the studies. Remarkably, the m7G modification has been shown to be dynamic, and its function has been linked to the regulation of translation [45].

The question of whether m7G exists in other classes of RNA remains controversial. Borohydride reduction sequencing (BoRed-seq) has been used to show that miRNA let-7 is methylated by METTL1 and the installed 7-methylguanosine is vital for the biogenesis of this miRNA. However, these findings have been disputed [223], and although the authors of the original study responded to the criticism [224], another study utilizing a different method termed m7G mutational profiling sequencing (m7G-MaP-seq) also failed to detect any m7G sites in human miRNA as well as snRNA and snoRNA [46].

### 4.12. tRNA Structural Modifications

The largest fraction of RNA modifications in our selection were primarily implicated in structural roles in tRNA, where they maintained the correct folding and stability of the transfer RNA molecules and the desired parameters of the codon–anticodon interactions [225].

Modifications of tRNA in the anticodon region, primarily in position 34, are widespread because they are responsible for the mechanism of wobble base pairing, which allows one tRNA to recognize multiple mRNA codons, thus reducing the overall number of tRNAs required for translation [226]. For example, 5-methylaminomethyl-2-thiouridine (mnm5s2U) at this position is required for the proper decoding of the lysine-encoding codons AAA and AAG by tRNA^Lys3^, while another uridine modification, 5-methoxycarbonylmethyl-2-thiouridine (mcm5s2U), is essential for the proper decoding of NNR codons [88].

Bases adjacent to the anticodon are also frequently modified to alter the specific parameters of translation. Two modified adenosines, N6-isopentenyladenosine (i6A) and N6-threonylcarbamoyladenosine (t6A) found in position 37 directly adjacent to the anticodon, positively affected the translation efficiency and prevented frameshifting [111,112]. Another modification of the 37th residue, 1-methylguanosine, is always present in a subset of tRNA in all domains of life and has a critical function to prevent frameshifting [80]. Wyosine and its related modification wybutosine are complex modifications to guanosine that are synthesized by a group of enzymes [115]. They are found at position 37 in tRNA^Phe^ ubiquitously in archaea and eukaryotes, and they ensure effective codon recognition by stabilizing the codon–anticodon interactions during decoding on the ribosome [90]. Interestingly, some modifications in this region can restrict the recognition of certain wobble codons; for instance, this is the role of 2-thiocytidine (s2C) at position 32 in bacterial and archaeal tRNA^Arg^ and tRNA^Ser2^ [106]. This demonstrates the complicated functional interplay between modifications of tRNA, of which there are other examples [225]. The 32nd residue in tRNA is subject to another frequent modification in eukaryotic cells, 3-methylcytosine. While the exact function of this modification is not yet known, its disruption has been associated with impaired cellular growth and oncogenesis [96].

Another set of modifications affects other regions of tRNA and usually performs structural functions. N2,N2-dimethylguanosine, which is almost always present in positions 10 and 26 of tRNA in archaea and eukaryotes, plays an essential role in the correct folding of tRNA [108]. Only one modification in our list is exclusive to archaea, and is suitably named archaeosine [102]. This modification is always found at position 15 and seldom in position 13 [103]. So far, the only known function of archaeosine in tRNA is its ability to increase the molecule’s thermostability, which may be especially important to archaea since many archaeal species are thermophiles [103].

Several modifications thought to be exclusive to tRNA were discovered in other RNA types, most notably mRNA. For instance, 1-methylguanosine and N2,N2-dimethylguanosine were identified in *S. cerevisiae* mRNA in small quantities using LC/MS-MS and were also shown to affect the translation efficiency in a position-specific manner [82]. However, further studies are required to confirm these results.

## 5. Conclusions and Perspectives

In recent years, there has been an increased interest in the epitranscriptome. Among the existing RNA modifications, currently numbering ~300 variants, special attention has been focused on a few of them, such as m6A, I, Ψ, m7G, and Nm. This is caused by their high frequency of occurrence in all domains and sequences, a wide range of functions performed, and several options for relatively simple detection methods.

Most RNA modifications are found in tRNA, and LC-MS/MS-based approaches are used for their detection. Sequencing-based methods are utilized mostly to detect modifications in other types of RNA molecules, in particular mRNA. Nowadays, protocols have been developed for processing data obtained using both nanopore-based long-read sequencing (direct RNA-seq) and more traditional short-read technology (RNA-seq).

The gap in the popularity of research on epitranscriptomic events is significant. Our analysis of epitranscriptome studies revealed that other modifications are mentioned in less than 20% of articles compared with the ubiquitous top five modifications (Table S1). This bias does not provide an opportunity to look extensively at the functions performed by the epitranscriptome. However, by discussing the currently known information about other modifications in the same context and in an equal manner, we illustrated the integrated complexity and interconnectedness of the epitranscriptome, therefore improving the recognition of less-studied modifications. This will hopefully lead to more active research efforts to investigate their properties in full detail.

Although their function is still unknown for most RNA modifications, it has been shown that in some cases, they play a key role in the development of a number of diseases and can be used as therapeutic targets. In particular, the study of the epitranscriptome initiated the development of mRNA vaccines. Although the first attempts did not reach the clinic, eventually, COVID-19 mRNA vaccines showed their high efficiency in preventing severe disease [227,228,229]. Such a success will stimulate interest in the development of RNA-based drugs. In addition, RNA modification modulators may serve as potential therapeutic targets for cancer. Nowadays, epitranscriptomic drugs have shown potential to promote the efficacy of chemotherapy in preclinical studies, and clinical trials should be designed and conducted in the future [230,231].

All of the above would have been impossible without the development of NGS technology, enabling it to detect modifications with a resolution of up to one nucleotide. However, not all RNA modifications can be detected by NGS-based methods, e.g., m3U and Q. The development of new experimental and bioinformatics methods will expand the list of modifications—“favorites”, paving the road for novel epitranscriptomic biomarkers, drugs, and vaccines.

In conclusion, we believe that the present review will improve the recognition of the lesser-known modifications, many of which are currently being found in additional classes of RNA and have already been shown to perform important biological functions. Considering the increasing evidence of the dynamic and interconnected behavior and regulation of these modifications, it is essential to investigate the full functional capacity of all, not just most, prevalent RNA modifications to comprehensively understand the epitranscriptome. Furthermore, the integration of the known information regarding specific properties of certain RNA modifications can lead to improved and more universal methods for their detection.

## Figures and Tables

**Table 1:** Available information on selected RNA modifications; the table is filtered by the frequency of occurrence of modifications in articles.

Modification	Code	Base	Domains	RNA Classes	Location and Frequency	Function	Methods	Diseases	Ref.
N6-methyladenosine	m6A	A	All	rRNA, tRNA, mRNA, miRNA, lncRNA, circRNA, snRNA	mRNA: enriched in 5′-UTRs, around stop codons, and in 3′ UTRs	mRNA: regulates splicing events, induces mRNA instability, increases translation efficiency; lncRNA: plays a role in lncRNA-mediated transcriptional repression	DRS, HPLC MeRIP-seq, MiCLIP	Osteoporosis, HCC, obesity	[21,22,23,24,25,26,27]
Inosine	I	A	All	tRNA, mRNA, miRNA	tRNA: p. 34, 37, 57; mRNA: dsRNA regions	tRNA: required for wobble codon recognition; mRNA: affects stability and localization, can change protein sequence	LC-MS/MS, RT-PCR, DRS, de novo RNA-seq	HCC; gastric, colorectal, esophageal, glioblastoma, and lung cancers	[28,29,30,31,32,33]
Pseudouridine	Ψ or Y	U	All	rRNA, tRNA, mRNA, snRNA, Mt-tRNA, scaRNA, snoRNA, miRNA, lincRNA	tRNA: p. 55; mRNA: 5′UTR, 3′UTR, CDS	rRNA: plays a role in ribosome assembly and translational fidelity; tRNA: increases stability	Pseudo-Seq, DRS, PSI-seq, CeU-Seq, Ψ-Seq, RBS-seq	Prostate, breast, and lung cancers; HCC	[34,35,36,37,38,39,40,41]
7-methylguanosine	m7G	G	All	miRNA, rRNA, mRNA, tRNA,	rRNA: SSU G1639 (in humans); tRNA: p. 46; mRNA: 4522 clusters within 2318 mRNAs in 293T cells	tRNA: increases stability; mRNA: regulates translation	m7G-MeRIP-seq, m7G-seq, DRS, m7G-miCLIP-seq, m7G-MaP-seq	HCC, PAD, lung cancer	[35,42,43,44,45,46,47,48]
Dihydrouridine	D	U	All	tRNA, mRNA, snoRNA	tRNA: p. 16, 17, 20, 20a, 20b, 47; mRNA: 130 sites in 112 transcripts in *S. cerevisiae*; snoRNA: 48 sites in 23 snoRNA in *S. cerevisiae*	tRNA: destabilizes the structure; mRNA: affects splicing and translation	D-seq, Rho-seq, LC-MS	Lung cancer	[49,50,51,52,53]
N1-methyladenosine	m1A	A	All	rRNA, tRNA, mRNA, lncRNA	rRNA: p.1322 in 28S; tRNA: p. 9, 14, 16, 22, 57, 58; mRNA: rare	rRNA: ensures proper folding; tRNA: ensures stability and proper folding	ARM-seq, m1A-seq, m1A-quant-seq, m1A-ID-seq, m1A-seq-SS, m1A-seq-TGIR, m1A-MAP, m1A-IP-seq	HCC; cervical, pancreatic, breast, and ovarian cancers	[17,54,55,56,57,58,59]
Queuosine	Q	G	Bacteria,eukaryotes	tRNA	tRNA^Tyr^, tRNA^His^, tRNA^Asn^, tRNA^Asp^: p. 34 (humans)	Impacts coding potential of tRNA, protects tRNA from ribonuclease cleavage	UHPLC-MS/MS, LC-MC	T-cell lymphoma, colon cancer	[60,61,62,63]
5-methylcytidine	m5C	C	All	rRNA, tRNA, mRNA, eRNA, miRNA, lncRNA, viral RNA, vault RNA, snRNA, snoRNA	rRNA: m5C3761 and m5C4413 (well-conserved in eukaryotes); tRNA: variable loop, anticodon loop, and T stem; mRNA: enriched in 5′UTR	rRNA: ensures proper folding and translational fidelity; tRNA: ensures proper folding and stability, codon–anticodon interactions, and reading frame maintenance; mRNA: affects stability, translation rate; lncRNA: affects stability; vault RNA: required for biogenesis	m5C-RIP-seq, Aza-IP-seq, miCLIP-seq, TAWO-seq, RNA-BisSeq, RBS-seq, DRS, MeRIP-seq	ARID, DS, lactic acidosis, breast cancer, hypotonia /floppy baby syndrome, metabolism	[14,40,48,64,65,66,67]
5-formylcytidine	f5C	C	All	tRNA	mt-tRNA^Met^: p.C34 (always)	Required for decoding of AUA codon in mitochondria	HPLC-MS	NA	[68,69]
5-methyluridine	m5U	U	All	rRNA, tRNA	tRNA: p. 54	Ensures fidelity of translation, folding, and stability of tRNA	FICC-Seq, iCLIP, miCLIP-seq	Breast cancer, systemic lupus erythematosus	[70,71,72,73]
2′-O-methylation	Nm	All	All	rRNA, tRNA, mRNA, snRNA, snoRNA	mRNA: 5′UTR, 3′UTR, CDS; tRNA: p. 4	Nm increases the thermodynamic stability of RNA:RNA base pairs and stabilizes A-form RNA duplexes	RiboMethSeq, 2D-TLC, Nm-seq, 2′OMe-seq, RTL-P, DRS, RibOxi-seq	Asthma, Alzheimer’s disease	[48,74,75,76,77,78,79]
1-methylguanosine	m1G	G	All	tRNA, mRNA	tRNA^Leu^_CUN_, tRNA^Pro^_CCN_, tRNA^Arg^_CGG_: p. 37 (always); tRNA: p. 9 (always in *S. cerevisiae*); mRNA (0.00046% G in *S. cerevisiae*)	tRNA: prevents frameshifting; mRNA: reduces translation fidelity in a position- and codon-dependent manner	MR-FISH, LC-MS/MS, DRS	NA	[80,81,82,83,84,85,86]
5-methylaminomethyl- 2-thiouridine	mnm5s2U	U	All	tRNA	tRNA^Lys3^_UUU_: p. 34 (always)	Required for decoding of the lysine codons AAA and AAG by tRNA^Lys3^_UUU_	LC-MS/MS	NA	[87,88,89]
Wybutosine	yW	G	Archaea, eukaryotes	tRNA	tRNA^Phe^: p. 37 (often)	Stabilizes codon–anticodon interactions	LC-MS/MS	NA	[90,91]
N4-acetylcytidine	ac4C	C	All	rRNA, tRNA, mRNA	tRNA: p. 12 (always); rRNA: in helix 34, 45; mRNA: enriched in 5′UTR and CDS (4250 sites in HeLa; not present in HEK293; 0.1% C in *S. cerevisiae* mRNA)	tRNA: increases translation efficiency and accuracy; mRNA: increases stability	acRIP-seq, ac4C-seq	NA	[92,93,94,95]
3-methylcytidine	m3C	C	Eukaryotes	tRNA	tRNA: p. 32 (30–90%, depending on tRNA)	Unknown	AlkAniline-seq, HAC-seq, ARM-seq, DM-tRNA-seq, mim-tRNA-seq, hydro-tRNA-seq, LC-MS/MS, NAIL-MS	Several types of cancer	[57,96,97,98,99,100,101]
Archaeosine	G+	G	Archaea	tRNA	tRNA: p. 15 (always), p.13 (in some species)	Increases thermal stability of tRNA	HPLC	NA	[102,103]
5-methoxycarbonylmethyl- 2-thiouridine	mcm5s2U	U	Eukaryotes	tRNA	tRNA^Lys^_UUU_, tRNA^Glu^_UUC_, tRNA^Gln^_UUG_: p. 34 (always)	Improves decoding efficiency of tRNA	HPLC-MS	NA	[88,104]
2-thiocytidine	s2C	C	Bacteria, eukaryotes	tRNA	tRNA^Arg^, tRNA^Ser2^_GCU_: p. 32 (always)	Restricts recognition of certain wobble codons	HPLC-MS	NA	[105,106]
N2,N2-dimethylguanosine	m2,2G	G	Archaea, eukaryotes	tRNA, mRNA	tRNA: p. 10, p. 26 (always); mRNA (0.00051% G in *S. cerevisiae*)	tRNA: plays a role in tRNA folding and prevents tRNA from adopting wrong conformation; mRNA: reduces translation fidelity in a position- and codon-dependent manner	PhOxi-seq, LC-MS/MS	NA	[82,107,108]
N6-isopentenyladenosine	i6A	A	Bacteria, eukaryotes	tRNA	tRNA_UNN_: p. A37 (always)	Increases translation fidelity and efficiency of cognate codons	PHA6 assay, HPLC, Sanger	NA	[109,110,111]
N6-threonylcarbamoyladenosine	t6A	A	All	tRNA	tRNA_ANN_: p. A37 (always)	Facilitates codon–anticodon pairing and prevents frameshift during protein synthesis	LC-MS/MS	MERFF, neurodegeneration, diabetes	[110,111,112]
3-methyluridine	m3U	U	All	rRNA, tRNA	rRNA: U1498 (always in *E. coli* 16S), U2634 U2843 (in *S. cerevisiae* 25S); tRNA^Thr^: p.U32 (always in *T. brucei*)	Unknown	MR-FISH, RP-HPLC	NA	[85,113,114]
Wyosine	imG	G	Archaea	tRNA	tRNA^Phe^: p. 37 (often)	Stabilizes codon–anticodon interactions	HPLC-MS	NA	[115,116]
1-methylinosine	m1I	I	Bacteria, archaea, eukaryotes	tRNA	tRNA^Ala^: p. 37 (eukaryotes); tRNA: p. 57 (archaea)	Unknown	LC-MS/MS	NA	[83,117]

NA—information not found; mRNA—messenger RNA; rRNA—ribosomal RNA; tRNA—transfer RNA; miRNA—microRNA; lncRNA—long noncoding RNA; snRNA—small nuclear RNA; snoRNA—small nucleolar RNA; eRNA—enhancer RNA; Mt-tRNA—mitochondrial tRNA; scaRNA—small Cajal body-specific RNA; lincRNA—long intergenic noncoding RNA; 5′-UTR—5′-untranslated region; 3′-UTR—3′-untranslated region; CDS—coding sequence; FMN—flavin mononucleotide; Dus—dihydrouridine synthase; LC-MS/MS—liquid chromatography–mass spectrometry; RP-HPLC—reversed-phase liquid chromatography–tandem mass spectrometry; MeRIP-seq—methylated RNA immunoprecipitation sequencing; miCLIP—methylated individual-nucleotide-resolution crosslinking and immunoprecipitation; PA-m6A-seq—photo-crosslinking-assisted m6A sequencing; m6A-CLIP—m6A individual-nucleotide-resolution crosslinking and immunoprecipitation; DRS—direct RNA-seq; HCC—hepatocellular carcinoma; MERFF—myoclonic epilepsy with ragged red fibers; PAD—peripheral arterial disease; ARID—autosomal recessive forms of intellectual disabilities; DS—Dubowitz syndrome.

## Supplementary Materials

The supporting information can be downloaded at: https://www.mdpi.com/article/10.3390/ijms232213851/s1.
